# The Neurocognitive Profile of the Cerebellum in Multiple Sclerosis

**DOI:** 10.3390/ijms160612185

**Published:** 2015-05-28

**Authors:** Alessia Sarica, Antonio Cerasa, Aldo Quattrone

**Affiliations:** 1 Istituto di Bioimmagini e Fisiologia Molecolare (IBFM), National Research Council (CNR), Catanzaro 88100, Italy; E-Mail: sarica@unicz.it; 2 Institute of Neurology, University Magna Graecia, Germaneto, Catanzaro 88100, Italy; E-Mail: quattrone@unicz.it

**Keywords:** cerebellum, multiple sclerosis (MS), magnetic resonance imaging (MRI), cognitive impairment, executive functions

## Abstract

In recent years, a high number of studies have demonstrated that neuropsychological functions are altered in multiple sclerosis (MS) patients with cerebellar lesions, mainly including attention, working memory and verbal fluency. Since the present literature is often elusive on this topic, we aim to provide a comprehensive report about the real impact of cerebellar damages (evaluated as volume, lesions or connectivity measures) on cognitive functions. In particular in this review, we report and discuss recent works from 2009 to 2015, which have demonstrated the key role of the cerebellum in cognitive impairment of MS patients.

## 1. Introduction

Multiple sclerosis (MS) is a common disease that affects the central nervous system (CNS), in which focal inflammation causes the degradation of myelin in the nerve fibers [1]. Beyond physical disability, cognitive impairment is recognized as a core feature of the disease, with prevalence estimates ranging from 40% to 65% [2]. Overall, the main cognitive functions affected by this disorder are: memory, processing speed, executive functions, attention and concentration and visuospatial abilities [3]; whereas other cognitive domains—such as language, short-term memory and general intelligence—are generally preserved [4].

The evaluation of cognitive impairment in MS has an important role since this has a dramatic impact on a patient’s quality of life, influencing role fulfillment in work, coping strategies and social life [5]. The most important cognitive functions associated with the loss of quality of life are: executive functions and memory. Executive functions are related to cognitive abilities that are needed especially for the adaptation to environment changes. Examples are the ability of planning, anticipating results and directing resources adequate to objectives and maintaining attention during long period under distractions of unfavorable conditions [6]. Recently, Goretti *et al.* [5] demonstrated that MS patients with lower performance in sustained attention were less prone to adopt positive coping strategies. Moreover, memory deficits influence the learning abilities that consist in the acquisition operations for formation, conservation and evocation of information [6]. It is usually classified into long and short duration working memory and operational memory, where: (i) short-term memory represents a sort of passive storage; (ii) long-term memory is related to the ability of learn new information and store them for unlimited time; (iii) operational memory refers to the capacity of storing important information, crucial for a complex task, for short periods (six/eight hours). In MS patients, memory and processing speed deficits can be worsened by depression, fatigue and motor disability [7].

Generally, the assessment of cognitive deficits is performed by comprehensive neuropsychological assessment batteries [8]. Unfortunately, neuropsychological assessment batteries are often very time consuming; therefore, Rao *et al.* [9] proposed the Brief Repeatable Battery of Neuropsychological Tests (BRB-N), which comprises the Buschke Selective Reminding Test (verbal learning and memory), the 7/24 Spatial Recall Test (visual learning and memory (SPART)), the Paced Auditory Serial Addition Test (working memory and resistance to interference (PASAT)), the Controlled Oral Word Association Test (verbal fluency and word retrieval (COWAT)), optionally, the Symbol Digit Modalities Test (processing speed and working memory (SDMT)) and the Stroop conflict task (measure of response modulation (ST)). BRB-N showed a high specificity and sensitivity and is one of the most employed batteries in this field of study. Again, the Minimal Assessment of Cognitive Function in MS (MACFIMS) [10] is another battery that, while minimizing the number of tests, covers all common deficits in MS: processing speed, working memory, learning and memory, executive functioning, visuospatial processing and word retrieval. The MACFIMS component tests are PASAT, SDMT, COWAT, California Verbal Learning Test-II (verbal learning and memory), Brief Visuospatial Memory Test-Revised (visual learning and memory), Delis-Kaplan Executive Function System (executive functioning and problem solving) and Judgment of Line Orientation (visuospatial processing (JLO)). Apart from batteries specifically designed for the assessment of cognitive impairment in MS, other neuropsychological tests are commonly employed: the Rey Osterrieth Complex Figure (ROCF) for visual perception and long term visual memory, the Rey Auditory Verbal Learning Test (RAVLT) for short-term auditory-verbal memory, the Word List Generation Test (WLG) for verbal fluency, the Paced Visual Serial Addition Test (PVSAT), a visual version of the PASAT, the Wisconsin Card Sorting Test (WCST) and its shortened version, the Modified Card Sorting Test (MCST) for the evaluation of executive functions and conflict resolution.

One of the main missions of neuroimaging is to define the neural substrates of these cognitive deficits in MS patients, and in the last few years the cerebellum has attracted much attention. Functional neuroimaging and lesion studies have corroborated the critical role of the cerebellum in cognition providing a detailed mapping of its involvement [11,12], mainly due to several tight anatomical connections with a number of higher-level cortical regions. In particular, the prefrontal cortex and the lateral parietal cortex show diffuse projections to different cerebellar regions via the thalamus and the pons [12,13,14,15,16,17,18]. In the neuropsychological realm, lesions in the cerebellum have produced the well-known “cerebellar cognitive affective syndrome” [19], a complex syndrome that includes executive dysfunctions and other cognitive deficits.

However, in MS patients the role of the cerebellum has not been well established. Indeed, despite that MS is traditionally considered as a white matter (WM) demyelinating disease, recent studies have demonstrated extensive involvement of gray matter structures and the cerebellum, already in early phases of the disease [20,21,22]. Moreover, the cerebellum has traditionally been viewed as purely involved in motor dysfunctions of MS patients, as demonstrated by studies [23,24] evaluating the clinical correlates of cerebellar signs. Indeed, gait ataxia, poor coordination of the hands, and intention tremor are usually the result of dysfunctionality in the cerebellum. These neurological signs are often reported in MS and it has been estimated that 30% of patients with Relapsing-Remitting Multiple Sclerosis (RR-MS) present at least one lesion within the cerebellum, while in roughly 11% of MS patients, the cerebellar symptoms and signs represent the predominant clinical manifestation [23]. Although the motor and affective consequences related to these neurological signs (*i.e.*, contributors to the progression of disease disability and impact on life quality) have widely been studied, their cognitive correlates are still unclear.

The goal of this mini-review was to report evidence provided by neuroimaging studies evaluating the cognitive profile of the cerebellum in MS patients mainly focusing on patients with cerebellar signs. Although, until now, few studies focused on this particular topic, important advances in the understanding of pathophysiological mechanisms of MS have been reached.

## 2. Results

As said in the Introduction, MS affects both motor and cognitive functions, and several studies have shown that a link exists between cerebellar dysfunction and specific cognitive impairment in MS. In this section we report those results that support this hypothesis, and we summarize eight structural and functional neuroimaging studies from 2009 to 2015. In particular, we investigated the correlation between cerebellar abnormalities and cognitive impairment as measured by neurophysiological tests. For this purpose and for facilitating comparisons, we summarized all relevant clinical, neuroimaging and neuropsychological findings in Table 1.

**Table 1:** Summary of works that found out a strong correlation between cerebellar abnormalities and cognitive impairment as measured by neuropsychological tests. For a list of abbreviation see Abbreviations section.

Article	MS Phenotype	No. of Subjects	Imaging Method	MRI Features	Neuropsychological Findings	Neuroimaging Findings
Valentino *et al.*, 2009	RR-MSc	21	Semi-automated morhpometry	Cerebellar and cortical TLL	RR-MSc: Deficits in SDMT and COWAT with respect to RR-MSnc	RR-MSc: No significant correlation between cognitive impairment and TLL
	RR-MSnc	21				
Cerasa *et al.*, 2012	RR-MSc	12	Semi-automated morhpometry/fMRI task	Cerebellar TLL and volume	RR-MSc: Deficits in SDMT, ST and WLG with respect to controls	RR-MSc: Cerebellar TLL correlated with fMRI activity of the superior parietal lobule during PVSAT
	RR-MSnc	15				
	Controls	16				
Cerasa *et al.*, 2013	RR-MSc	12	Semi-automated morhpometry/VBM	Cerebellar TLL and GM volume	RR-MSc: Deficits in SDMT, COWAT and ROCFT with respect to RR-MSnc and controls	RR-MSc: GM volume in the dorsolateral prefrontal cortex correlated with SDMT; GM volume in superior temporal gyrus correlated with COWAT
	RR-MSnc	14				
	Controls	20				
Damasceno *et al*., 2014	RR-MS	42	Semi-automated morhpometry/FreeSurfer	Cerebellar GM/WM volume, intracortical/leukocortical lesions (nr. and volume)	RR-MS: deficits in SDMT and PASAT with respect to controls	RR-MS: Cerebellar intracortical lesions associated with SDMT, Cerebellar leukocortical lesions associated with PASAT
	Controls	30				
Rocca *et al.*, 2014	RR-MS	121	fMRI connectivity	Global and regional network properties, hubs	–	RR-MS: PASAT correlated with global network features, with absence of additional hubs in Lobule VII of right cerebellum, and with a decrease of nodal degree in right cerebellum
	B-MS	45				
	SP-MS	80				
Romascano *et al.*, 2014	RR-MS	28	Automated morhpometry/fMRI connectivity	Cerebellar lesion (nr. and volume), CPN parameters: T1 rt, T2 rt, MTR, GFA	RR-MS: No cognitive deficit	RR-MS: T1 rt, T2 rt, GFA associated with the SRT-LTS, GFA correlated SRT-CLTR and SDMT, T2 rt correlated with SPART-D
	Controls	26				
Weier *et al.*, 2014	RR-MSc	120	Semi-automated morhpometry/SIENAX	TCV, CGV, CWV	RR-MSc: Deficits in SDMT and PASAT with respect to RR-MSnc	RR-MSc: TCV correlated with SDMT, cerebellar T1 lesion volume significant predictor of PASAT
	RR-MSnc	52				
Deppe *et al.*, 2015	RR-MS	68	DTI	Cerebellar FA, AD, MD, RD	*–*	RR-MS: Cerebellar FA correlated with motor disability scale (EDSS)
	Controls	26				

### 2.1. Valentino et al., 2009

In 2009, Valentino *et al.* [25] performed a study on a cohort of RR-MS patients with the aim of exploring differences in cognitive functions between subjects with (RR-MSc, 21 subjects) and without (RR-MSnc, 21 subjects) cerebellar damages. In particular, subjects performed a battery of neuropsychological tests and MRI scans have been acquired. The authors quantified the total lesion load (TLL) and furthermore they performed regional lesion load measurements, including four main brain areas: frontal, parietal, temporal and occipital lobe. All subjects showed evidence of cognitive impairment in specific domains, while all cerebellar patients (RR-MSc) had MRI evidence of lesions in the cerebellum or in the cerebellar pedunculi. Both RR-MSc and RR-MSnc groups presented the greatest lesion load in the frontal lobe and the smallest in the occipital lobe. The main finding was that RR-MS patients with cerebellar symptoms performed worse in the areas of attention (SDMT) and verbal fluency (COWAT) than individually matched MS patients without cerebellar damage. However, regional lesion loads did not affect the detected cognitive profile of RR-MSc patients.

### 2.2. Cerasa et al., 2012

Cerasa *et al.* [26], similarly to Valentino *et al.* [25], were interested in investigating the neurofunctional influence of cerebellar signs on MS patients. They studied two MS groups with (12 subjects) and without cerebellar symptoms (15 subjects) compared with healthy controls (16 subjects) who underwent a functional MRI study (fMRI). Since the previous neuropsychological paper [25] demonstrated the presence of executive dysfunctions in RR-MSc patients, functional connectivity analysis was performed during the execution of a well-known working memory fMRI task (PVSAT), widely employed to assess the functional integrity of the parieto-prefrontal network. In agreement with Valentino *et al.*, 2009, the authors found that RR-MSc patients displayed statistically significant low performances in attention/working memory (SDMT), abstract reasoning (ST) and verbal fluency (Word List Generation (WLG)). This specific cognitive pattern was not related to the magnitude of cerebellar lesion load as measured on Fluid Attenuated Inversion Recovery (FLAIR) images. Functional connectivity analysis demonstrated that during the fMRI PVSAT task, RR-MSc patients were characterized by an abnormally reduced functional connectivity between the left cerebellar Crus I and the right superior parietal lobule (BA 7) when compared with either RR-MSnc patients or controls. Differing from neuropsychological evidence, the detected dysfunctional pattern in RR-MSc patients was correlated with the measurement of the cerebellar lesion load. In other words, the increasing of cerebellar damage correlated with enhanced activity of the superior parietal lobule.

### 2.3. Cerasa et al., 2013

In an extension of the previous work, Cerasa *et al.* [27] corroborated their results by investigating the presence of neuroanatomical abnormalities in a population of RR-MSc patients (12 subjects) compared them to RR-MSnc patients (14 subjects) and controls (20 subjects). All patients completed neuropsychological assessments and (FLAIR) axial images were used for calculating hyperintense lesion volumes. At a neuropsychological level, the authors reconfirmed the selective cognitive impairment in RR-MSc patients was characterized by lower performance in attention (SDMT), verbal fluency (COWAT) and spatial memory tests (ROCFT) either with respect to RR-MSnc patients or to controls. Advanced structural neuroimaging analysis demonstrated that, with respect to controls, RR-MSnc patients were characterized by a specific atrophy of the bilateral thalami that became more widespread (including motor cortex) in the RR-MSc group. Moreover, the RR-MSc group showed atrophies in the prefrontal and temporal cortical areas when directly compared with the RR-MSnc group. Gray matter volume losses in these two cortical regions were also related to poor performance in SDMT and COWAT, respectively.

### 2.4. Damasceno et al., 2014

Damasceno *et al.* [28] conducted a study to evaluate the influence of cerebellar pathology studying 42 RR-MS patients compared with 30 healthy controls. In particular, they aimed at evaluating the influence of lesions in the cerebellum—such as intracortical and leukocortical—on the clinical and cognitive outcomes of RR-MS patients. Brain white matter lesion load (WML), brain cortical lesions, presence of cerebellar WM lesions and cerebellar gray matter (GM) lesions were identified. As a result, cerebellar intracortical and/or leukocortical lesions and cerebellar WM lesions were observed in more than a half of the patients. Although patients with high loads of cerebellar leukocortical lesions had similar scores on clinical outcomes compared to those with lower burdens of these lesions, patients with high burdens of cerebellar intracortical lesions showed worse performance in motor clinical evaluations. Furthermore, when Damasceno *et al.* discriminated between cerebellar intracortical and leukocortical lesions, they found that intracortical lesions correlated with clinical dysfunction and SDMT performance, while leukocortical lesions correlated with the PASAT scores. Thus, Damasceno *et al.* proved that cerebellar GM lesions are partially involved in clinical and cognitive disability related to MS.

### 2.5. Rocca et al., 2014

Rocca *et al.* [29] graphed a theoretical analysis on MS patients (246 subjects) and controls (55 subjects) in order to explore the topological organization of functional brain network connectivity. This kind of analysis was applied to resting state fMRI data with the aim of estimating the functional connectivity between 116 cortical and subcortical brain regions, using a bivariate correlation analysis. Several global network properties proved to be abnormal in MS patients *versus* controls, such as: (i) loss of hubs in the superior frontal gyrus, precuneus and anterior cingulum in the left hemisphere; (ii) different lateralization of basal ganglia hubs and (iii) decreased nodal degree in the bilateral caudate nucleus and right cerebellum. Next, the authors focused their attention on the link between several brain areas: they suggested that the redistribution of functional hubs can be “functionally ineffective or associated with more severe clinical manifestations”, as in the case of hubs located in the cerebellum and lingual gyrus in cognitively impaired patients. Patients were considered cognitively impaired (CI) when the PASAT score was 2SD below the average score of a comparable control group. Following this sub-division of groups, the authors found that CI patients had lower values of global network features (mean network degree, global efficiency and hierarchy) and a higher path length than cognitively preserved patients (CP) and controls. Regarding regional network properties, when compared to CP patients, the CI patients did not present additional hubs in the lobule VIII of the right cerebellum, but they did show a decreased nodal degree in the right cerebellum (Crus I and lobule VIII). More generally, an impaired functional connectivity between the cerebellum and the frontal lobes has been shown to contribute to failure of cognitive compensation in MS subjects.

### 2.6. Romascano et al., 2014

Romascano *et al.* [30] used a new multicontrast connectometry approach to assess the structural and functional integrity of cerebellar networks and connectivity in MS. The authors enrolled 28 RR-MS subjects and 16 healthy controls and acquired diffusion spectrum imaging (DSI) and resting-state fMRI (rs-fMRI). These scans were used to construct structural and functional cerebellar connectomes, while quantitative MRI relaxometry and magnetization transfer imaging (MTI) (multicontrast connectometry) were used to investigate axonal degeneration, micro-inflammation and demyelination. Imaging data were then correlated with clinical tests assessing disability, motor and cognitive functions. Although multicontrast cerebellar connectometry found no changes in structural network organization properties or functional connectivity, it revealed subtle local connectivity disruptions in a group of early MS patients. Furthermore, local connectivity alterations, which indicated loss of axonal integrity and tissue microstructure, were highly correlated with motor and cognitive function in MS patients. In particular, the cerebellar network showed several local structural alterations in links of cerebellar lobules, strictly correlated with prevalent motor and cognitive (*i.e.*, SDMT) functions.

### 2.7. Weier et al., 2014

Weier *et al.* [31] focused their attention on the relationship between cerebellar volumes, clinical cerebellar signs, cognitive functioning and fatigue in MS patients. They analyzed the total cerebellar volume (TCV), the cerebellar gray matter volume (CGV) and the white matter volume (CWV) of 172 MS patients. The link between these features and cognitive functioning (SDMT, PASAT) were investigated by a hierarchical multiple linear regression analysis. The authors confirmed previous findings, demonstrating that MS patients with cerebellar signs had more cognitive decline with respect to others in PASAT and SDMT. Moreover, they found that this cognitive profile is predicted by specific MRI-related measures. In particular, the normalized total cerebellar volume is a significant predictor of SDMT performance, while cerebellar T1 lesion volume can significantly predict PASAT decline.

### 2.8. Deppe et al., 2015

Deppe *et al.* [32] investigated a large group of RR-MS (65) without cerebellar signs, compared to 26 controls, with the aim to identify subtle cerebellar microstructural alterations in patients with RR-MS, even in patients without relevant obvious infratentorial lesions visible by conventional MRI. To do that these authors employed Diffusion Tensor Imaging (DTI) metrics (including fractional anisotropy (FA)), measured in the whole-brain and in the cerebellum. Deppe *et al.* revealed cerebellar FA reduction in MS groups when compared to controls. Furthermore, cerebellar FA reduction was correlated with the Expanded Disability Status Scale (EDSS), disease duration, suggesting that cerebellar microstructural alterations are closely associated with the severity of the disease. This study demonstrated that DTI abnormalities could even be detected in patients without any cerebellar signs and thus that microstructural changes could represent a significant biomarker for the progression of MS. Unfortunately, cognitive correlates of these findings have not been reported.

## 3. Discussion

Overall, what clearly emerged from this brief review is that the cerebellum has a critical and specific involvement in the cognitive decline of MS patients. Either using a clinical model of cerebellar damage (MS patients with cerebellar signs) or generally investigating MS patients, it is evident that performance mainly related to executive functions is strictly related to the functionality and the integrity of the cerebellum. This neuropsychological pattern has also been confirmed by previous lesion studies demonstrating that focal cerebellar lesions *per se* lead to specific cognitive deficits in executive functions and language domains [12,33].

The links between cerebellar dysfunction and cognitive impairment in MS can be explained in many ways. One link is related to the anatomical connections between the neocerebellum and associated cortical areas, including the prefrontal cortex [16]. In particular—as demonstrated by Cerasa *et al.*, 2012 [26] and Rocca *et al.*, 2014 [29]—the hyperactivity of Crus I (lobule VI and VII) is mirrored by a dysfunctional connectivity with critical cortical regions. Cerasa *et al.*, 2012 found altered communication between Crus I and the superior parietal lobule during the execution of a working memory task (PVSAT). Rocca *et al.*, 2014 came to similar conclusions demonstrating that MS patients with cognitive impairment are characterized by loss of brain hubs in the Crus I region. Interestingly, the definition of cognitive impairment was based upon the presence of cognitive deficits in PASAT—a broadly used task in MS populations that requires a high involvement of the posterior “cognitive” cerebellum, including the Crus I. This region is involved in many cognitive functions including language, visuospatial skills and working memory [11,34]. As concerns working memory performance, it has been proposed that its involvement is specifically related to the articulatory control system [35,36]. Indeed, Baddeley’s [35] model proposed a framework for working memory called the phonological loop, which consists of two components: a phonological short-term store, which can hold speech-related information for 1–2 s, and an articulatory control system, which serves to sub-vocally refresh the contents of the phonological store. After a series of functional MRI experiments [37,38,39], it has been proposed that Crus I is part of the cortico-subcortical circuit (also including Broca’s area) that is related to the articulatory control system [35,37]. Therefore, cerebellar overactivation in MS patients may represent an increased effort to subvocally refresh phonological stimuli in an articulatory control system. Another important observations are pointing to the possibility that some cognitive dysfunctions in MS patients are predicted by atrophy and lesion load within the cerebellum. This evidence has been described in some papers reported in this mini-review (Valentino *et al.*, 2014, Cerasa *et al.*, 2012, Damasceno *et al.*, 2014, Romascano *et al.*, 2014 and Weier *et al.*, 2014), although with discrepancies. Damasceno *et al.*, 2014 found that cerebellar lesions are more predictive of cognitive deficits in executive functions (as measured by PASAT and SDMT) with respect to volumetric measurements of the cerebellum. Romascano *et al.*, 2014—using a different neuroimaging approach—confirmed that cerebellar volumetry is moderately related to clinical outcome, while cerebellar lesions are strictly related to both clinical and cognitive scores (including SDMT performance). Moreover, Weier *et al.* found that both cerebellar lesion and atrophy in MS patients with cerebellar signs predicted the cognitive performance during SDMT and PASAT. However, Cerasa *et al.*, 2009 and Cerasa *et al.*, 2013 reported opposite findings—that cognitive impairment related to attention and language domains in MS patients with cerebellar signs is not dependent upon either cerebellar lesions (measured as total lesion load) or cerebellar atrophies. Instead, they found that lower cognitive performance is related to gray matter atrophies in specific cortical areas strictly connected with the cerebellum: the dorsolateral prefrontal cortex (for SDMT deficits) and temporal cortex (for COWAT performance). In Deppe *et al.*, 2015, no neuropsychological tests were performed, but we have taken into account this work because this is the first demonstrating that DTI-related metrics of the cerebellar cortex might represent a more sensitive and reliable marker of microstructural changes occurring, especially, in early stage of MS. To the best of our knowledge, no DTI studies had clearly evaluated the relationship between microstructural WM changes in the cerebellum and cognitive dys-functioning in MS patients.

With regards to the discrepancies among findings here presented, they might be due to several aspects. One aspect is related to the study sample—the small number of subjects, the different phenotypes considered and the enrollment procedure. Another aspect is the employment of different MRI acquisition techniques and feature extraction methods—lesion load quantification (manual or semi-automated), voxel-based morphometry or connectometry approaches. The final aspect, even if less important, is the statistical procedure for analyzing data that must be considered, since univariate and multivariate approaches can lead to very different estimates.

## 4. Conclusions

The purpose of this mini-review is to summarize and compare recent studies evaluating the impact of cerebellar damage on cognitive impairment in MS. Indeed, MS is an extremely heterogeneous disorder both at the clinical and pathological level. In particular, while several cortical and sub-cortical regions can be affected, the precise relationships between cognitive function and MS damage remain elusive. Hence, determining more precisely the intimate link between specific brain circuits and distinct cognitive profiles may be an important advancement for future refinements of MS diagnosis [26]. A better characterization of the cognitive profiles in MS may also impact the future developing of neurobiological markers aimed at tracking the disease progression, with clear implications for the prognosis and treatments (*i.e.*, cognitive rehabilitation [40]).

The recent neuropsychological and neuroimaging literature strongly supports the view that executive dysfunctions in MS are strongly dependent upon cerebellar functioning. Clinically speaking, this finding confirms that patients with cerebellar damages may define a distinct clinical subtype. However, modern neuroimaging is not yet fully able to provide a reliable biomarker of this relationship. In other words, it has not been clearly defined which MRI-related metric (lesion load, lesion volume, volumetric GM quantification, microstructural WM changes as detected by DTI) better represents the neurodegenerative processes underlying cerebellar-related cognitive decline in MS. Further studies employing multimodal imaging approach are strongly needed to address this fundamental topic.

## Abbreviations

9HPT: 9-Hole Peg Test
B-MS: Benign MS
BRB-N: Brief Repeatable Battery of Neuropsychological Tests
COWAT: Controlled Oral Word Association Test
DR: Delayed Recall
DTI: Diffusion Tensor Imaging
EDSS: Expanded Disability Status Scale
FA: Fractional Anisotropy
FSS: Fatigue Severity Scale
GFA: Generalized Fractional Anisotropy
GM: Grey Matter
IR: Immediate Recall
JLO: Judgment Line Orientation
MCST CA: Modified Card Sorting Test Categories Achieved
MCST PE: Modified Card Sorting Test Perseverative Errors
MMSE: Mini Mental State Examination
MS: Multiple Sclerosis
PASAT: Paced Auditory Serial Addition Task
PP-MS: Primary Progressive MS
PVSAT: Paced Visual Serial Addition Test
RAVLT: Rey Auditory-Verbal Learning Test
Rec: Recognition of lists of words
ROCFT: Rey-Osterrieth Complex Figure Test
RR-MSc: Relapsing-remitting MS patients with cerebellar symptoms
RR-MSnc: Relapsing-remitting MS patients without cerebellar symptoms
SDMT: Symbol Digit Modalities Test
SP-MS: Secondary Progressive MS
SPART-I: Spatial Recall Test-Immediate
SPART-D: Spatial Recall Test-Delayed
SRT-LTS: Selective Reminding Test-Long Term Storage
SRT-CLTR: Selective Reminding Test-Consistent Long Term Storage
SRT-D: Selective Reminding Test-Delayed
ST: Stroop task
T25FW: Timed-25 Foot Walk test
T2LL: T2-hyperintense lesion load
TCV: Total Cerebellar Volume
CGV: Cerebellar Grey matter Volume
CWV: Cerebellar White matter Volume
TLL: Total Lesion Load
VBM: Voxel-based Morphometry
WCST: Wisconsin Card Sorting Test
WLG: Word List Generation test
WM: White Matter
